# The International Cancer Expert Corps: A Unique Approach for Sustainable Cancer Care in Low and Lower-Middle Income Countries

**DOI:** 10.3389/fonc.2014.00333

**Published:** 2014-11-19

**Authors:** C. Norman Coleman, Silvia C. Formenti, Tim R. Williams, Daniel G. Petereit, Khee C. Soo, John Wong, Nelson Chao, Lawrence N. Shulman, Surbhi Grover, Ian Magrath, Stephen Hahn, Fei-Fei Liu, Theodore DeWeese, Samir N. Khleif, Michael Steinberg, Lawrence Roth, David A. Pistenmaa, Richard R. Love, Majid Mohiuddin, Bhadrasain Vikram

**Affiliations:** ^1^International Cancer Expert Corps, Chevy Chase, MD, USA; ^2^New York University Medical School, New York, NY, USA; ^3^Lynn Cancer Institute at Boca Raton Regional Hospital, Boca Raton, FL, USA; ^4^American Indian “Walking Forward” Program, Rapid City, SD, USA; ^5^National Cancer Center, Singapore, Singapore; ^6^National University Cancer Institute, National University of Singapore, Singapore, Singapore; ^7^Division of Hematologic Malignancies and Cellular Therapy, BMT and Global Cancer, Duke Cancer Institute, Duke University, Durham, NC, USA; ^8^Dana-Farber Cancer Institute, Harvard Medical School, Boston, MA, USA; ^9^Department of Radiation Oncology, Abramson Cancer Center, University of Pennsylvania, Philadelphia, PA, USA; ^10^International Network for Cancer Treatment and Research, Brussels, Belgium; ^11^Department of Radiation Oncology, Radiation Medicine Program, Princess Margaret Cancer Centre, University of Toronto, Toronto, ON, Canada; ^12^Department of Radiation Oncology and Molecular Radiation Sciences, Johns Hopkins University, Baltimore, MD, USA; ^13^Cancer Center, Georgia Regents University, Augusta, GA, USA; ^14^Department of Radiation Oncology, University of California Los Angeles, Los Angeles, CA, USA; ^15^International Breast Cancer Research Foundation, Madison, WI, USA; ^16^Radiation Oncology Consultants, Ltd., Park Ridge, IL, USA; ^17^Radiation Research Program, National Cancer Institute, Bethesda, MD, USA

**Keywords:** health disparities, cancer, global health, underserved, non-communicable diseases

## Abstract

The growing burden of non-communicable diseases including cancer in low- and lower-middle income countries (LMICs) and in geographic-access limited settings within resource-rich countries requires effective and sustainable solutions. The International Cancer Expert Corps (ICEC) is pioneering a novel global mentorship–partnership model to address workforce capability and capacity within cancer disparities regions built on the requirement for local investment in personnel and infrastructure. Radiation oncology will be a key component given its efficacy for cure even for the advanced stages of disease often encountered and for palliation. The goal for an ICEC Center within these health disparities settings is to develop and retain a high-quality sustainable workforce who can provide the best possible cancer care, conduct research, and become a regional center of excellence. The ICEC Center can also serve as a focal point for economic, social, and healthcare system improvement. ICEC is establishing teams of Experts with expertise to mentor in the broad range of subjects required to establish and sustain cancer care programs. The Hubs are cancer centers or other groups and professional societies in resource-rich settings that will comprise the global infrastructure coordinated by ICEC Central. A transformational tenet of ICEC is that altruistic, human-service activity should be an integral part of a healthcare career. To achieve a critical mass of mentors ICEC is working with three groups: academia, private practice, and senior mentors/retirees. While in-kind support will be important, ICEC seeks support for the career time dedicated to this activity through grants, government support, industry, and philanthropy. Providing care for people with cancer in LMICs has been a recalcitrant problem. The alarming increase in the global burden of cancer in LMICs underscores the urgency and makes this an opportune time fornovel and sustainable solutions to transform cancer care globally.

## Introduction

The growing burden of non-communicable diseases (NCDs) in the developing world has been highlighted by the World Health Organization (WHO) report in 2010 and in a United Nations (UN) declaration in 2012 (1, 2). Love et al. (3) have proposed the concept of public health oncology, which describes the multiple levels of complexity for addressing the problems of delivery of cancer care. It emphasizes that cancer and the other NCDs are embedded in economic, social, political, gender, healthcare, and public health issues. The discussion of NCDs does not reduce the ongoing importance of the communicable diseases but, in fact, highlights the broad spectrum of diseases now encountered globally.

Coleman and Love have addressed the need for a transformative approach to science, service, and society, emphasizing that the task of reducing the burden of disease among health disparities populations is arguably as integral a component of academic translational medicine as are laboratory and clinically based research (4). This current paper describes the organizational structure and operational approach of an international collaborative organization, the International Cancer Expert Corps (ICEC). ICEC uses a unique mentorship model to help develop and sustain a workforce within cancer health disparities setting who are capable of conducting multi-modality cancer care and research at international standards. While healthcare disparities are well known to exist in lower-middle income countries (LMICs), similar problems also occur in resource-rich countries where people have difficulty accessing cancer care as a result of poverty, cultural issues, limited economic opportunity, and geographic distance from a cancer treatment center^1^. Considering a frequently expressed question “Why is the focus of ICEC on international when there are domestic problems?” ICEC recognizes that there are indeed common problems and potentially similar solutions among LMICs and geographic-access limited settings within resource-rich countries. In particular, the latter involve significant numbers of “aboriginal” or native populations, so that ICEC will address the geographically access limited issue in resource-rich countries as a global problem, which will benefit from the lessons learned from international collaboration. The LMIC community will provide the local investment in personnel and necessary infrastructure with whom the ICEC will provide mentorship. It is recognized that these are significant challenges for resource-poor communities; nonetheless, local buy-in and support are deemed to be critical to a sustainable program.

Partnering with and enhancing ongoing global health programs is an essential tenet of ICEC. Given the ICEC focus on mentoring and workforce development, collaboration with existing efforts will be mutually beneficial. Potential collaborating organizations include (a) international agencies such as the Union for International Cancer Control (5) and its Global Task Force on Radiotherapy for Cancer Control (6) and the International Atomic Energy Agency’s program of action for cancer treatment (PACT) (7); (b) research focused governmental institutions such as the Center for Global Health in the National Cancer Institute (8); (c) oncology professional societies from various countries and specialties, (d) oncology projects between resource-rich and resource-limited settings, including “twinning” projects (9) between academic centers and facilities within a LMIC setting such as those of Partners-in-Health (10) and AMPATH (11), and (e) international collaborative programs for education and research including the International Network for Cancer Treatment and Research (INCTR) (12).

## Methods

The development of the model for ICEC is the result of decades of experience of a number of the authors from working in the underserved communities in the U.S. and globally. Examples include the PACT (7) and INCTR (12) programs mentioned above, the Harvard community outreach program in Massachusetts (13), the Cancer Disparities Research Partnership Program of the National Cancer Institute (14), the Walking Forward Program in South Dakota (15), experience in breast cancer care in Bangladesh (16), and the establishment of King Hussein Cancer Center Program as a major cancer program through shared expertise between Jordan and the NCI (17). The recent emphasis on non-communicable disease burden in global health (1, 2) led to recognition of the need for innovative approaches to healthcare. This is accompanied by unprecedented opportunity across a number of economic, healthcare, social, and political sectors (4). Building on ongoing discussions among global colleagues, including experts at the National Institutes of Health Fogarty International Center (18) and Center for Global Health at NCI (8), the ICEC model continues to take shape as ICEC moves into implementation. Key underpinnings are that ICEC is a multi-national global effort at the outset and that it is taking on a very difficult challenge for which innovation and sustainability are deemed to be essential.

The ICEC has seven essential characteristics for a science-grounded strategy:

1. Decrease cancer incidence and mortality and improve quality of life globally. Use specific benchmarks and defined metrics to assess all interventions.
2. Build an international effort from the outset with collaboration across countries, sectors, and disciplines.
3. Emphasize local initiative built from community leaders or “champions.” The projects will be established from the bottom-up based on local needs and opportunities coupled with the ability of ICEC to help leverage local investment. ICEC will not build physical infrastructure.
4. Establish research efforts including implementation science (defined in Section “Research”) and programs across the cancer control spectrum from prevention to treatment to follow-up to elucidating mechanisms of cancer biology.
5. Aim for the availability of effective treatments including cure and palliation for every patient with cancer in the world within the next two decades. This is in concert with the Global Health 2035 goals (19).
6. Develop sustainable worldwide capacity and capability through public health approaches, applications of innovative economic and business models, greater knowledge sharing, and exploiting new information technologies.
7. Work to effect a cultural change that values and rewards as an integral part of a career the efforts for working on human-service efforts.

Notably, this approach to cancer is applicable to NCDs in general. The public health and systems approach is consistent with that described by Kim (20), which focuses on HIV/AIDS emphasizing a systems level analysis and interventions across the healthcare system involving broader social and economic determinants of health. This is similar to the issues described in public health oncology by Love (3). The ICEC career approach helps meet the objectives of an international service corps described by Kerry (21) and builds on the suggestion that the more classic components of academic healthcare careers, research, education, and patient care, which have already been broadened in scope in the last few decades further expand the academic mission to include policy, social responsibility, and service to addressing overarching societal issues, including health disparities (22).

## Results

### International cancer expert corps

#### Description and vision

The ICEC is an international mentoring network of cancer professionals who will work with local and regional in-country groups on projects to develop and sustain expertise and local solutions for better cancer care. The vision is a world in which everyone has access to cost-effective interventions to prevent and treat cancer and its symptoms in ways that are consistent with best possible practices for the local circumstances. Addressing and realizing this vision can benefit people everywhere because of the scientific, humanitarian, and diplomatic consequences of such projects.

#### Intervention model

A major issue in global health is whether national policy prescriptive approaches such as cancer plans, which are top-down efforts, should be the priority, or whether bottom-up, local community specific approaches are more likely to achieve our developmental goals over the long term (23). We believe aspects of both approaches are useful for building local capacity and capability, but the uncertainties about *how* to address the breadth of complex psychosocial–medical cancer issues indicate a need for more investigative-research driven, bottom-up efforts (3).

Therefore, the ICEC model is to establish LMIC programs from the inside out and from the bottom-up. The focus is on *people* and on sustainable mentoring and collaborative relationships among ICEC Experts and local Associates within ICEC Centers in regions/countries that will invest in solutions for the underserved. As detailed below, senior mentors and retirees will not only mentor Associates but also guide and mentor junior and mid-career faculty from resource-rich countries who aim to pursue a career path in global health. Mentoring will be accomplished by international teams of Experts whose goal is to apply guideline- and protocol-driven care at international quality standards and be capable of joining international research groups as they so choose. The product of this relationship will be cancer programs in cancer disparities settings with the capacity, capability and credibility to (a) assume a leadership role in their own region; (b) bring new knowledge and approaches to addressing cancer disparities issues globally; and (c) be equal partners among the world expert cancer educators and researchers.

#### Organizational structure

**Figure 1:**
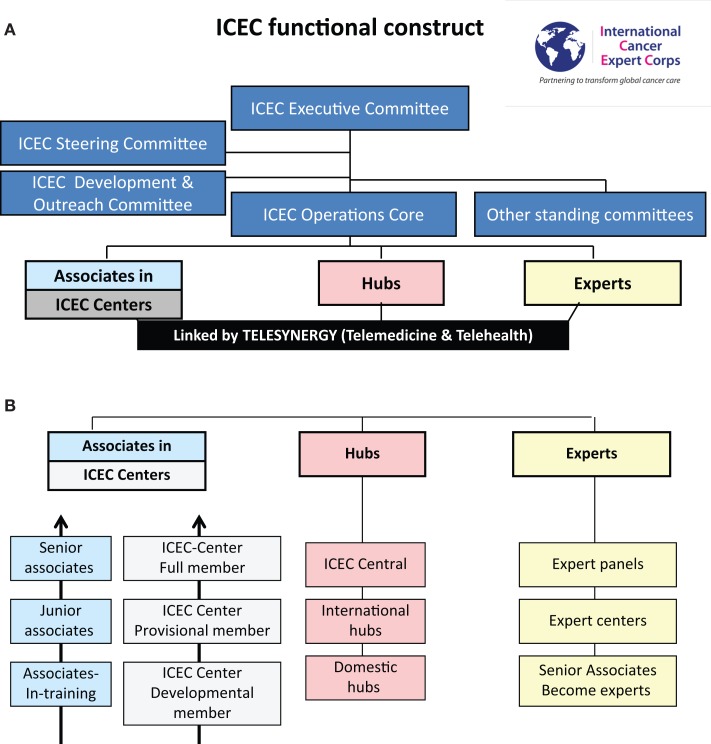
**(A)** ICEC functional construct, **(B)** ICEC functional components.

Figure 1A illustrates the ICEC functional construct and Figure 1B includes ICEC functional components. The focus of the ICEC is to develop expertise in ICEC centers under the guidance of local ICEC Associates. The Centers will be linked to the ICEC through a Hub in their region. Hubs are cancer centers or other groups and professional societies in resource-rich settings that will comprise the global infrastructure coordinated by ICEC Central. While visits between Experts and Centers are critical, mentoring will largely be accomplished by scheduled teleconferences to teach and review multi-modality care through guidelines and protocols.

The focus of the ICEC is to work with *Associates* who are local change-makers or “champions” serving health disparities populations. They will work in medical facilities/locations where there is a multi-year commitment to investing in infrastructure and people toward improving the quality of care and life for their affected citizens. The facilities are designated *ICEC Centers*. Using a multi-year, jointly prepared “bottom-up” plan, the Associates progress from Associate-in-training to Junior Associate to Senior Associate based on defined metrics. The Center will progress from a Developmental Member to Provisional Member to (Full) Member, which requires passing a “cooperative group” quality site visit. Once Senior Associate and Full Member status are achieved the Center can then serve as a Hub for their region.

**Figure 2:**
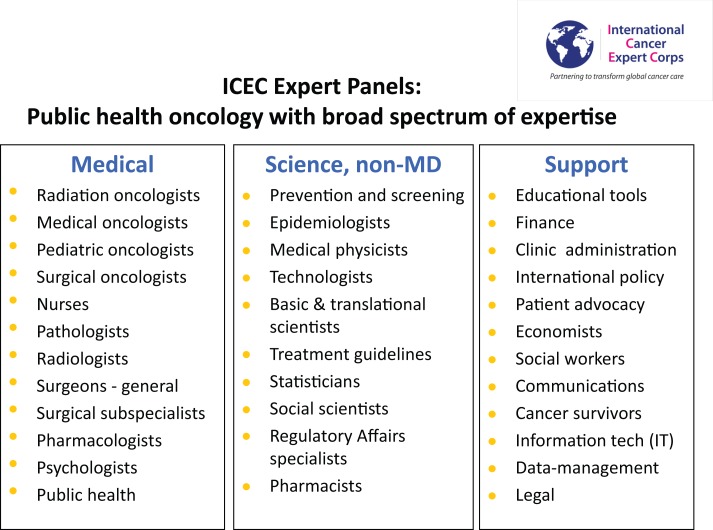
**ICEC expert panels**. A broad range of expertise is required, although there will initially be a focused effort. Expertise is required in the standard medical disciplines for cancer care, scientific, and medical disciplines for research and supporting disciplines to address the economic, societal, social, and political issues the comprise public health oncology (3).

*Experts* will include the range of oncology disciplines, healthcare delivery services and public health, economics, and policy specialists. The categories of the Expert Panels defined are in Figure 2. Multiple ICEC experts will mentor an Associate/ICEC Center in the conduct of guideline- and protocol-based care and not on individual patient management. To ensure sustainability, there will be a required commitment of time and effort for the Experts. Experts include senior expert academicians and mentors, private practitioners, faculty, and trainees in the range of academic ranks from institutions who will help design and support a formal career path for human service. Experts may join as individuals, institutions, societies, or teams.

*Hubs* provide the infrastructure and, working through ICEC Central, will coordinate the linkage between the Associate/ICEC Center with the Expert mentor so that the professional time is spent on mentoring and education. By having resource-rich Hubs worldwide share the “on call” duty there can be essentially full-time person-to-person connectivity and highly efficient coordination of time and use of resources. Hubs will be major academic cancer centers, private practices, professional societies, and others. While ICEC is worldwide and all Hubs have a global focus, some Hubs that focus primarily on LMICs will be international hubs and those that address health disparities geographic-access issues within resource-rich countries, such as the native populations in the US, Canada, and others, will be domestic Hubs recognizing that their health disparities issues have solutions in common with those of LMICs.

#### Building partnerships

**Figure 3:**
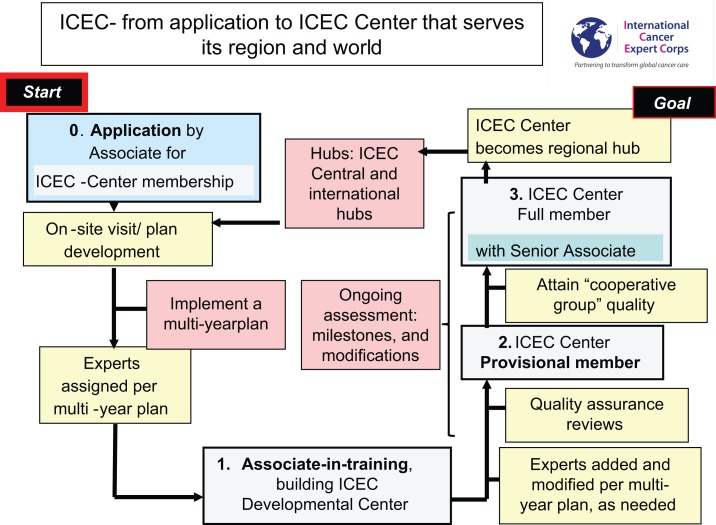
**Progression from application to Full Member and Senior Associate**.

The process for how a facility or group within a health disparities region will work with ICEC to become an ICEC Center is illustrated in Figure 3.

An application review will help determine the composition of the initial team from ICEC to meet on-site with the applicant. This on-site discussion includes a needs assessment and exchange of ideas that will help ICEC and Associate/Center develop a mutually acceptable multi-year plan. The new ICEC Developmental Center will be paired with a regional Hub. The initial ICEC Experts will be assigned from throughout the global network based on the initial needs of the Center. The Associate serving as the Principal Investigator Associate (may be Associate-in-training or possibly already more senior) and the team at their ICEC Developmental Center will begin the process of establishing multi-modality cancer care. This will likely be a multi-year process, possibly up to 5 or so years. There will be occasional visits among the Associate/Center and Experts, coordinated by their regional Hub, but mentoring will be primarily accomplished through scheduled teleconferences for “case” reviews for the patients who are being treated on the specific guidelines or protocols, which are being used for the mentoring and training. The Associate and Center will progress in capability for cancer management to where an initial Quality Assurance Site Visit is passed. Further program development and mentoring will involve some of the initial Experts for continuity and also the addition of others with the growing scope of expertise in the Center.

Ongoing evaluation of progress for all components of ICEC is essential for guiding development and to learn from experience. It is anticipated that formal research will be conducted that will range from implementation science to translational research to clinical trials to social and economic research. At a point in time when there is multi-modality care, data-management systems, and the ability to adhere to guideline- or protocol-based care a “clinical cooperative group” site visit will be passed, which indicates that the ICEC Center is ready to apply for full participation in worldwide clinical trials. ICEC is not an accreditation body so that approval of the ICEC Center’s participation in such studies would be the responsibility of the particular research program or agency. Once (a) the ICEC site visit is passed and a level of expertise achieved, (b) there is a Senior Associate as program leader and other Associates as members, and (c) the Center is a Full Member, the ICEC Center could become a regional Hub for ICEC.

### Career path

#### Careers that include global health

Education and training are key activities of ICEC. It is expected that the Associates are completing or will have undergone formal training in their discipline, although it is recognized that the extent of training and specific credentials will vary. As noted above, a breadth of expertise will be required to mentor physicians, nurses, scientists, epidemiologists, and other healthcare and health policy workers from LMICs in public health oncology (3), and in global health. A key aspect of ICEC is that the Associates in LMICs can provide care and also are trained to critically analyze local cancer care systems and develop approaches for improvement. The latter will be shared among ICEC and published so that others might benefit from lessons learned. Annual meetings at different locales will enhance cross-cultural education and sharing of ideas and experience.

Given its central role in treatment and cure of malignancy often encountered at advanced stage in LMIC and in its palliative potential, radiation therapy will be a requirement. If not present at the outset there must be a clear plan and timeline to obtain this capability within the first few years. Establishing radiation therapy, diagnostic imaging, and laboratory capabilities in settings that may not have stable infrastructure (power, water, communications, etc.) provides an enormous opportunity for technological research and development, creating affordable treatment paradigms, and developing novel approaches for remote-access medicine and means of utilizing and deploying information technology. While ICEC will not supply equipment, we will bring together industry partners and economists to (a) address the need for appropriate technology, including cobalt units, brachytherapy, imaging, including basic CT and linear accelerators possibly of novel modular design so that complexity of treatment will progress as skills develop; (b) investigate sustainable economic models for affordable treatment with a goal of a course of cancer treatment for approximately $400, the approximate cost of a cataract operation in LMICs and in line with the challenge by Kerr (24); (c) examine business models that not only help fill the enormous cancer care gap and open new markets (25) but also potentially cluster facilities so that regional service centers are economically viable; and (d) develop a skilled workforce connected to international expertise who will be able to utilize the technology safely and effectively.

#### Career in global health is needed for sustainability

The current career paths in academia involve clinical, laboratory, and translational research, education, public health/outcomes research, and patient care. While global health is being emphasized in undergraduate education and to some extent in training, it remains an area for substantial academic exploration since at present a very limited number of people are engaged in this aspect of healthcare as a routine component of their career. To that end, we believe that there is the need for a transformational approach to return this type of altruistic service to where it is an integral component of a healthcare career (4) and not a side light.

This requires pioneering institutions to create a *bona fide* career path in academia for healthcare service to the underserved by providing an organizational and academic base in resource-rich centers of excellence for public health oncology experts (26). This would involve enhancing the focus of global health programs from their current emphases on general training of medical students to emphases on service and research that can be maintained throughout faculty careers as are laboratory and translational research, teaching, and clinical care. The current value system in healthcare would be modified by providing time and academic recognition for this type of activity to further emphasize values of social responsibility and service. Allocating time and establishing a new value and reward system for altruistic service may have positive ramifications on mitigating spiraling healthcare expenditures (13, 27).

#### Global resource and expertise sharing

**Figure 4:**
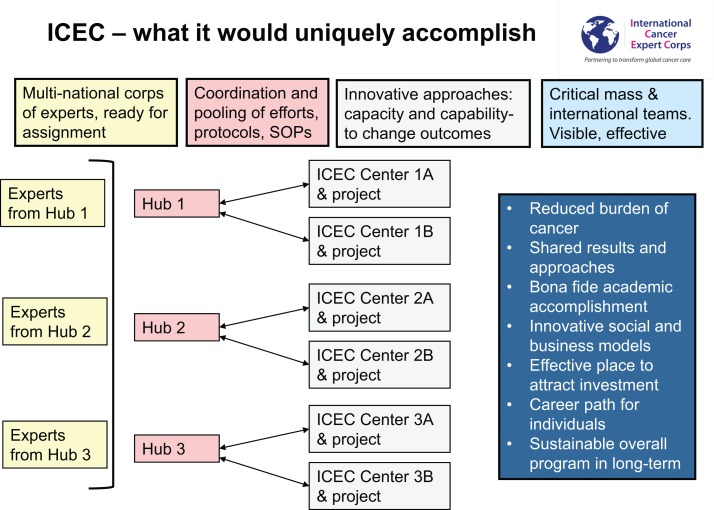
**Global outreach based on local investment and collaboration**.

Figure 4 summarizes the global interconnectivity of the ICEC model. ICEC Centers will link to the network through a Hub in their region. Coordinated through ICEC Central operations (*vide infra*) the Hub would call on Experts who are reliably available because they have time and effort predictably committed to this activity. To enhance the global nature of cultural interchange and idea sharing, a Center and Associate will have Experts that come from different countries within the global network. Senior mentors will mentor Associates and also educate early-career Experts who thereby gain access to world renowned mentors. Critically, work and family lives do not need to be disrupted for extended periods of time. Because the great majority of the time for mentoring is in scheduled educational “case” conferences (akin to radiation oncology chart rounds), the ICEC designated time can be part of a standard career in academia and practice. Educational materials developed by professional societies, the International Atomic Energy Agency and its virtual university for cancer control (VUCnet) (28), and others will be utilized to avoid duplication of efforts.

The unique aspect of ICEC is the assembling of a critical mass of global health expertise. There are already “twinning” programs among academic centers and facilities in LMICs and international programs with whom to partner and enhance breadth and depth. Sharing of expertise and resources means that the investment by any one Hub is not excessive while the system-wide aggregate is substantial.

As it now stands many twinning programs between resource-rich cancer centers and LMICs depend on the efforts of a few people. By having programs work together and share ideas, models, expertise, and resources, a robust networking system can be created that can have continuity and sustainability beyond a founder. The four boxes on the top of the figure describe what will be done while the box on the right side includes the long-term goals.

### ICEC operations

#### ICEC operations

There is an increasing interest in global health attested by the rapid growth in the consortium of universities for global health (CUGH) (29), a partner with ICEC. Many of the programs are either short or medium-term visits with no or limited follow-up. For those of us creating ICEC, it became evident that sustainability is essential with the ability to make decisions when opportunities arise. We concluded that sustainability is best achieved within a not-for-profit, non-government organization that can partner readily with government agencies and work across international boundaries. ICEC accomplishes this by having Hubs and Centers established locally that collaborate through facile central coordination, agreed-upon standard operating procedure and guidelines, and by sharing resources through mechanisms such as memoranda of understanding, contracts, grants, and other agreements. Mutual goals, addressing important problems, and trust are important components of mentoring, innovation, and growth. For the ICEC systems approach vision, organization, planning, execution, and adaptability are essential.

**Figure 5:**
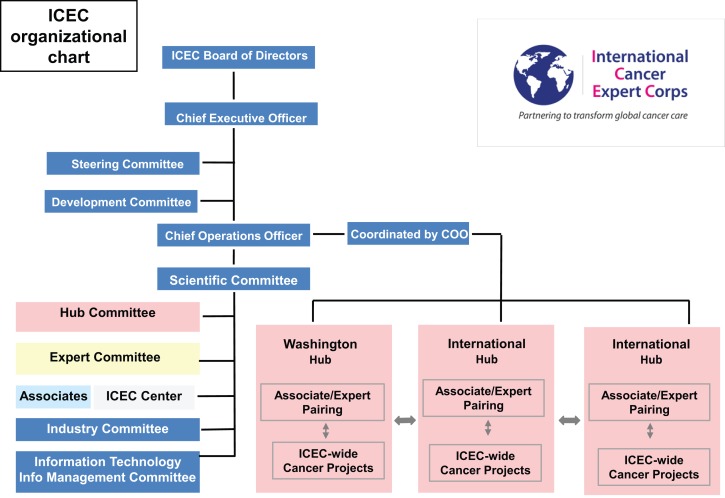
**Organizational chart**. The structure and terms of office are detailed in the business plan and by-laws. There is a Board of Directors overseeing the organization. A Steering Committee is a subset of the Board active in the detailed management. A Board of Advisors will provide input to the Board of Directors. The functions of and relationships among the hubs, experts, associates, and centers are described above including Figure 1. See text below for detailed discussion of ICEC operations.

Figure 5 is a detailed organizational chart for ICEC. As with any complex system, attention to details, assessing progress, and outcomes, and experience-based flexibility are critical. It is recognized that the structure and function will evolve.

Each expert panel in Figure 2 will consist of a leader and members, the goal of which is to have at least 20% of each Expert’s time committed (8 h/week, on average over the year) to ICEC efforts as a *bona fide* component of their job. The full-time equivalent (FTE) concept is used so that five people contributing 20% of their time would be one FTE. In the start-up of ICEC, there will be a limited number of ICEC Centers/Associates, Hubs, and Experts. Some of the initial groups are below, recognizing there will be rapid expansion of breadth of Expert panels:

Initial diseases (and the public health problem and oncologic opportunity included)

- Cervix (implementation of standard external beam and brachytherapy services, sexually transmitted disease, vaccine);
- Head and neck [smoking, combined modality therapy with radiation plus chemotherapy using cost-effective drugs (applicable in other cancers)];
- Lung (respiratory diseases, potential for hypo-fractionated (few-fraction) radiotherapy and novel combined modality therapy with radiation plus chemotherapy);
- Breast (women’s issues, screening, genetic disease), hypofractionation, breast brachytherapy for early stage disease;
- Palliative care (reduce burden of care on families and healthcare system and reduce suffering, immobility, and potential abandonment for patient);
- Lymphoma (relates to a younger population and for which collaborative programs are in place).

Initial panels (there will be other Experts as needed for diseases above)

- Radiation oncology
- Medical oncology
- Surgical oncology
- Palliative care physicians
- Medical physics, technology (including industry to develop new technology)
- Nursing (anticipated to be a key underpinning of Centers)
- Data management and Information Technology (using cell phone technology)
- Imaging (including teleradiology- basic radiology and CT)
- Pathology (including telepathology)
- Pharmacists (especially for palliative care and cost-effective chemotherapy).

#### Development and outreach

The overarching development goal for ICEC is to provide partial salary support in the form of contracts/grants to enable altruistic service and global health to become an integral part of the spectrum of academic and professional careers. In that the goal is 20% of time, or 8 h/week on average over the year, ICEC will aim to have a matching program of ICEC support and in-kind contribution (equal match) thereby leveraging one funded ICEC FTE up to 10 Experts. The cost of any FTE supported will be based on the local pay scale with maximum limits set by the Board. (This will be at most the NIH FTE rate for resource-rich countries). Financial support for a position will make this career path possible, especially so in the changing face of healthcare; however, as critical or even more so than compensation is the career recognition and reward, which in academia includes promotion in rank, professional recognition and career advancement.

A unique aspect of ICEC will be drawing expertise from three tracks, each of which has untapped potential:

- *Academia*. A career path will proceed from trainee to junior faculty (Assistant Professor) to mid-career (Associate Professor) to senior faculty (Full Professor). As it now stands, students and trainees are often engaged in global health but there is not a well-defined or supported career path beyond training (26). ICEC is working with visionary leaders in formulating an approach toward a formal career path that can serve as a template for other interested universities.
- *Private practice*. During the initial presentations of ICEC it became clear that private practitioners have the keen interest, clinical skills, experience, and flexibility to serve as leaders and mentors. At the time of this publication, there are two practice groups in the United States that will be pilot Hubs. ICEC will help develop an advancement scheme that provides appropriate recognition for the individual contributions in a manner similar to that of academia.
- *Retirees*. With the major oncology societies approximately 50 years old and radiation and medical oncology specialty boards approximately 40 years old, there is now a growing cohort of senior mentors and retirees whose experience, wisdom, and interest in serving will be tapped. Having new challenges and opportunities for senior people will allow those interested to extend their careers and also to open up senior opportunities to junior faculty in their home department. They will mentor Associates and also younger Experts. With their international reputations they become role models for altruistic service and global health as a sustainable career path. In that much of their costs will be in-kind, this may help develop a novel economic model for healthcare, as suggested by Christensen (27) in that some of the more expensive “solution shops” (27) can be obtained at greatly reduced cost thereby substantially enhancing the value that ICEC brings to solving the underserved problem.

### Research

The ICEC conducts and enables research. Mentoring will help build capacity but there is much to learn about how to solve the economic and access problems of reaching the underserved and establishing the best treatments for their resource settings. Therefore, having capability to do research and accrue credible data, there is ample opportunity for the Associates and Centers to perform different types of research in addition to the more standard clinical trials. Some examples are as follows:

- *Implementation science*. As defined by the Fogarty International Center of the National Institutes of Health (30):
  Implementation science is the study of methods to promote the integration of research findings and evidence into healthcare policy and practice. It seeks to understand the behavior of healthcare professionals and other stakeholders as a key variable in the sustainable uptake, adoption, and implementation of evidence-based interventions.
  As a newly emerging field, the definition of implementation science and the type of research it encompasses may vary according setting and sponsor. However, the intent of implementation science and related research is to investigate and address major bottlenecks (e.g., social, behavioral, economic, management) that impede effective implementation, test new approaches to improve health programming, as well as determine a causal relationship between the intervention and its impact.
  The ICEC is addressing a problem that is unsolved and growing – cancer care in LMICs. It is piloting a complex system solution using collaboration, mentoring, and idea-sharing among countries, cultures, and disciplines that has transformational potential. High-quality data yielded from research will inform the evolution of this challenging process.
- *Translational research*. The opportunity to study unique aspects of cancer biology including infectious and environmental causes can expand the understanding of cancer and generate new treatments. This type of research can be initiated in the early phases of ICEC Center and Associate development and can provide immediate local benefit. Having options to access research may serve as an additional motivation for the Center to establish quality data management so that they can derive further benefits from new knowledge and also bring a level of prestige and respect that can enhance investment in their Center.
- *Economics, healthcare models*. Crisp, Christensen, Love (3, 27, 31), and others emphasize that the solution to addressing cancer and NCDs requires novel economic models. The breadth of Experts in Supporting Disciplines (Figure 2) will help develop sustainable solutions through new models and a collaborative network. This includes bringing in complex technology with the needed supporting services (e.g., maintenance, supplies, and technicians) and also using novel technology to simplify care (e.g., cell phones). Given the magnitude of the shortfall of resources the ICEC approach is amenable to using a pre-competitive, collaborative approach among industry to improve outcomes and to greatly expand markets for a range of goods and services.
- *Role for radiation oncology*. A recent series of articles organized by Zeitman (32) addresses the potential role and responsibility of radiation oncology for global cancer health. Datta and colleagues (33) provide a detailed description of the infrastructure and human resources shortages using data from GLOBOCAN, International Agency for Research on Cancer. Suggested remedies include capacity building, networking, and a challenge to industry for low-cost, affordable, low-maintenance equipment. Fisher and colleagues (34) and Page and colleagues (35) discuss the shortages in Africa and the pros and cons of cobalt and linear accelerators, both of which have roles. Fisher has pioneered a program Radiation Hope (36), which aims to obtain equipment and implement treatment (37).

A key to establishing sustainable programs and to the ICEC model is support from professional societies. There is a clear interest in global education by the American Society of Radiation Oncology (38) and the Association of Residents in Radiation Oncology (39). ICEC will aim to capitalize on this interest to where it can be a sustainable career path.

### Global network coordination and public-private partnership

#### ICEC central

The various components of ICEC will be coordinated by ICEC Central with policies and procedures developed by ICEC Committees (Figure 4). ICEC daily activities are conducted under the Chief Executive Officer, the Executive Secretary to the CEO, and the Chief Operating Officer with advice from the Senior Scientific Advisor. A Steering Committee of the Board of Directors is readily available as needed and will be involved in routine discussions with the Operations team. While ICEC is in start-up mode, individuals may assume more than one role. Working with the Board of Directors and Board of Advisors, the various committees are establishing the policies and procedures to be used throughout ICEC.

Initial committees are

- Experts and Application/Career Path
- ICEC Centers and Associates
- Hubs
- Operations/Information Technology-Information Management
- Scientific – which will consist of representatives from Experts, ICEC Centers and Associates and Hubs to determine research directions
- Industry – who will work with industry, including a pre-competitive model, to develop equipment and approaches for bringing technology and care to cancer health disparities populations
- Outreach and Development.

#### Non-government organization and public–private partnership

The ICEC is a non-government organization incorporated in the State of Delaware, United States and recognized the Internal Revenue Service as a 501 (c) 3 tax exempt entity. Given its primary focus of patient care, the mission of the ICEC is complementary to that of the Center for Global Health of the National Cancer Institute (8). To the extent permissible by federal regulations, ICEC will partner with the federal agencies.

## Discussion

A recent assessment of investment in global health pointed out diseases that cause the highest burden as measured by disability-adjusted life years (DALYs) do not get much of the international investment. The NCDs produce approximately 45% of the DALYs but receive <5% of the aid (40). Using the measure of the years of life lost (YLL), NCDs are a substantial problem starting with the 15–49-year-old age group and becoming the major cause of YLL for those age 50 and over, yet the development assistance for health (DAH) for NCDs is merely 1% of the total DAH in LMICs (41). The 2010 WHO global status report (1) and a related 2012 UN declaration (2) brought attention to the growing burden of NCDs including cancer in LMICs. Notably, case burdens are also increasing in rural underserved areas within resource-rich countries with the native/aboriginal populations often having similar access to care, poverty, economic, and social challenges as encountered in LMICs (15). Thus, for health disparities populations worldwide, cancer is a progressively urgent problem from medical, health system, business, workforce, economic, and ethical perspectives. A sustainable approach to build local capability and capacity is warranted. Cancer affects people in both resource-rich and resource-poor settings and serves as a compelling common global problem upon which to build partnerships and to develop novel highly collaborative sustainable approaches.

### Building on success and lessons observed

In addressing issues of global healthcare, Nigel Crisp suggested that critical premises for an ideal model include an understanding of the societies in which these occur focusing on public health with community and outpatient-centered services, building locally defined solutions with reliance on local skills (31). Christensen’s analysis of the failure of high-income country business models in health has provided three perspectives for a different “disruptive” roadmap for innovation in cancer health: the need for technological enabling, business model innovation and value networks (27). Yunus’ social business model is one upon which cancer care activities might be structured for sustainability and growth (42). These ideas all provide intellectual bases for the kinds of cancer health projects and economic and social investigation the ICEC will pursue, specifically building on the experience and assessment from those in the local setting, in addition to clinical care and research relevant to their situation. ICEC recognizes the importance of establishing metrics to assess programs and progress in order to justify ongoing investment (40). ICEC will build on the strong research culture in cancer care and on the proven outreach experience of the ICEC Hubs, the Cancer Disparities Research Partnership program from the National Cancer Institute (14), experience from the International Network of Cancer Treatment and Research (12), and collaboration with the NCI Center for Global Health (8, 43).

### Altruism in medicine

Healthcare expenditures continue to grow with economic models dominating how care is delivered and how professional compensation is determined. Perhaps not sufficiently part of the discussion and solution, observers have suggested that altruism is declining in medicine (36–38, 44–46). However, human service and altruism continue to be important aspects of a physician’s professional responsibilities and attitudes (26, 47), and these professionals are willing to give their time and efforts toward altruistic causes (48) a trend that appears to be growing amongst young people entering careers in medicine. However, “altruism cannot thrive due to its lack of rewards and feedback, particularly in the economic climate of today’s science” (48). Programs to effectively address issues of such importance as changing the course of global health can only be reliably sustained when such activities are an integral part of daily work. We believe that while the central skills of academic medicine remain clinical care, research, teaching, education, and mentoring, twenty-first-century responsibilities include public health, policy, and solving major societal problems and must be built on social responsibility and service (4, 22).

The establishment of ICEC itself is implementation science and ICEC will enable research to be conducted by and for the benefit of those in health disparities regions, which include those in resource-rich countries. Metrics will be established and appropriately modified based on experience to assess progress, develop novel strategies and share experience among the network of global partners. The breadth and depth of ICEC will be such that individual programs in LMICs are not dependent on single individuals so that long-term investment by the local community, industry and committed individuals has a high probability of success. We believe that the current crisis can no longer be ignored and that “it is too hard or too big a problem” are not acceptable answers. To quote Nelson Mandela: “It always seems impossible until it’s done” (49). For scientific, medical diplomacy, ethical and humanitarian reasons, the time is right for a major initiative to address cancer in LMICs. ICEC welcomes participation.

## Conflict of Interest Statement

The authors declare that the research was conducted in the absence of any commercial or financial relationships that could be construed as a potential conflict of interest.

## Footnotes

^1^These are referred to “geographic-access limited” in this paper that also includes cultural issues, poverty, and limited economic opportunity.
